# Eye-Hand Coordination during Visuomotor Adaptation with Different Rotation Angles

**DOI:** 10.1371/journal.pone.0109819

**Published:** 2014-10-15

**Authors:** Sebastian Rentsch, Miya K. Rand

**Affiliations:** Leibniz Research Centre for Working Environment and Human Factors (IfADo), Dortmund, Germany; VU University Amsterdam, Netherlands

## Abstract

This study examined adaptive changes of eye-hand coordination during a visuomotor rotation task. Young adults made aiming movements to targets on a horizontal plane, while looking at the rotated feedback (cursor) of hand movements on a monitor. To vary the task difficulty, three rotation angles (30°, 75°, and 150°) were tested in three groups. All groups shortened hand movement time and trajectory length with practice. However, control strategies used were different among groups. The 30° group used proportionately more implicit adjustments of hand movements than other groups. The 75° group used more on-line feedback control, whereas the 150° group used explicit strategic adjustments. Regarding eye-hand coordination, timing of gaze shift to the target was gradually changed with practice from the late to early phase of hand movements in all groups, indicating an emerging gaze-anchoring behavior. Gaze locations prior to the gaze anchoring were also modified with practice from the cursor vicinity to an area between the starting position and the target. Reflecting various task difficulties, these changes occurred fastest in the 30° group, followed by the 75° group. The 150° group persisted in gazing at the cursor vicinity. These results suggest that the function of gaze control during visuomotor adaptation changes from a reactive control for exploring the relation between cursor and hand movements to a predictive control for guiding the hand to the task goal. That gaze-anchoring behavior emerged in all groups despite various control strategies indicates a generality of this adaptive pattern for eye-hand coordination in goal-directed actions.

## Introduction

The accurate spatio-temporal coordination of eye and hand movements is important for skilled manual actions as it functionally allows effective sensorimotor processing for their planning and execution. In aiming movements to a target, initiation of saccades usually precedes that of hand movements [1]–[4], and the timings of these initiations are moderately correlated [5]–[9]. Such eye-hand coordination provides a functional advantage for the limbmotor system to improve reaching accuracy by perceiving the target in a foveal vision to update the planning of on-going reaching movement [1], [2], [10], [11]. Processing of retinal and extraretinal information due to gazing at the target also improves planning and control of aiming movements both for the ballistic phase and the feedback control phase [11]–[13]. Another well-known feature of the saccades during manual aiming movements is gaze anchoring, where the gaze remains fixated to a target until hand movement is completed [8], [9], [14]–[16]. This gaze pattern likely allows for effective use of on-line visual feedback of both the hand and the target during a homing-in phase to guide the hand to the target. This gaze behavior is also used to verify the completion of hand movements [8]–[9].

In humans, these typical patterns of eye-hand coordination are established through extensive practice in daily life. However, the processes of establishing the coordination patterns are not well understood. A previous study by Sailer et al. [17] on such learning processes during a novel sensorimotor transformation showed that gazes typically went across a large area in the working space including locations of visual feedback and the target at the early stage of learning. This gaze pattern helped to explore the relationship between manual actions to the target and resulting changes of visual feedback. In the well learned stage, gazes shifted directly to the target and stayed there until the feedback cursor arrived, thereby guiding the hands to bring the cursor to that target, namely guiding the hands to the task goal.

The study by Sailer et al. [17], however, required learning of a highly complex, novel sensorimotor mapping rule between visual input (i.e., feedback cursor) and motor output (i.e., complex bimanual hand manipulations involving isometric force productions and wrist rotations). Thus, learning-related changes observed in that study may not be generalized to more traditional tasks of visuomotor transformation where goal-directed reaching movements to targets are made under altered visual feedback in terms of amplitudes and/or directions of movements [18]–[23]. In such tasks, the trajectories of reaching movements to the targets before applying a visuomotor transformation are nearly straight, because the control of the freedom degrees of the muscular-skeletal system utilized for performing reaching movements is already optimized through daily life [24]–[26]. That means that a basic visuomotor mapping rule is already in place. In contrast, the task by Sailer et al. required participants to discover a novel rule to control the freedom degrees of muscular-skeletal system utilized for performing movements that bring a feedback cursor to the targets. Therefore, the traditional tasks involve adaptation of an existing visuomotor mapping rule rather than a discovery of a novel rule; and hence, the element of exploration to discover a basic mapping rule is smaller compared to the task by Sailer et al. [17]. Consequently, the gaze anchoring behavior may not be disrupted by an altered visuomotor map and would be maintained throughout the practice period. To our knowledge, such adaptive processes of eye-hand coordination have not been studied previously. Therefore, the current study investigates them by using a visuomotor rotation task.

In previous studies, the difficulty of visuomotor rotation tasks depended on the rotational magnitude of visual feedback [18], [27]–[29]. Small rotation angles resulted in short performance times and small behavioral errors between the target and the cursor. The values of these parameters increased as the rotation angle increased up to 90–120°. Hence, the larger the rotation angle was, the more difficult the task became. Thereafter, these values decreased as the rotation angle increased up to 180°, indicating that the adaptation was getting easier. Furthermore, it was suggested that adaptation to rotation angles probably up to 90°–120° involved a single rotational transformation, whereas that near 180° involved a two-step rotational transformation [18], [27], [29]. The first step was a relatively easy 180° polarity inversion of both axes (called as a reversal shift, [18]), and the second step was a “backward” shift to the rotated visuomotor map. Moreover, explicit knowledge of a visuomotor rotation was acquired through practice when the rotation was sufficiently large [30]–[33] but not when it was small due to a gradual increase of the rotation [32]–[34]. These observations suggest that not only the task difficulty but also the underlying adaptive processes may be different depending on the magnitude of visuomotor rotations [35]–[36]. This, in turn, raises a possibility that eye-hand coordination patterns during adaptation to visuomotor rotations also may differ depending on the rotational magnitude.

The purpose of the present study was to investigate adaptive changes of eye-hand coordination under different rotation angles of a visuomotor rotation task. Based on the study by Sailer et al. [17], one hypothesis was that gaze locations during the hand movements are variable across the work space in the early practice phase, but are stabilized on the target in the late practice phase to guide the hand to the target. Thus, a gaze anchoring behavior is gradually established through adaptation. An alternative hypothesis was that the gaze anchoring to the target is maintained throughout the practice period of a visuomotor rotation. That would imply that the control of already established gaze anchoring is independent from adaptive processes related to a visuomotor rotation. The present experiment utilized three rotation angles (30°, 75°, and 150°) to alter the difficulty of the visuomotor transformation. As stated above, the 30° angle is expected to be easier than the 75° angle, whereas the 150° angle is easier than 75°. Adaptation to the 30° and 75° rotations involves one rotational transformation, whereas that to the 150° rotation involves the two-step transformation. Accordingly, the involvement of a reversal shift makes the difference between the 150° and 30° rotations. Since an increased difficulty of aiming movements is known to enhance the involvement of a visual feedback control during movement execution [37]–[39], it was predicted that gazes would be more directed to a feedback cursor of the hand movement for more difficult transformations. Consequently, the establishment of gaze anchoring to the target for these transformations would be delayed during practice.

## Methods

### Participants

Thirty healthy young adults (mean ± SD: 24.0±2.9 years old, 15 females and 15 males) provided written informed consent and participated in the study. All participants were self-reported right handers. They were free of neurological or sensory impairments based on self-reporting. All participants had normal color vision according to the Ishihara test [40] and normal visual acuity. This study was approved by the ethics committee of the Leibniz Research Centre of Working Environment and Human Factors. The participants were randomly divided into three experimental groups with a visuomotor rotation task of 30°, 75° or 150° (10 participants each).

### Apparatus

Participants were comfortably seated on a height-adjustable chair in front of a table, on which a digitizer (Wacom Intuos 4XL, active area size: 488×305 mm) and a vertical 22-inch computer monitor (Dell P2210, flash rate: 60 Hz) were placed. An infrared eye-tracking system (iViewX 500 RED, SMI) was attached underneath the monitor. The participants rested their chin on a chin rest. The distance between participants' eye position and the eye tracker was 660±12 [SD] mm on average. A starting position (SP, black circles, 6 mm in diameter) displayed in the center of the monitor was aligned with the participants' median plane. Participants held a stylus with their right hand in a manner like holding a pen for handwriting and moved their hand in a horizontal plane on the digitizer. Visual feedback of hand movements was displayed as a cursor (a red circle, 4 mm in diameter) on the monitor. The maximum display delay of the visual feedback was approximately 37 ms. Movements of the stylus on the digitizer and those of the cursor on the monitor had a one-to-one ratio with respect to distance. Visual feedback of hand movements was displayed continuously during movements. An opaque board placed 170 mm above the digitizer surface blocked participants' view of their hand movements.

It should be noted that visual feedback was displayed in the vertical plane and hand movements were made in the horizontal plane under the current setting. Hence, participants had to deal with the tool's kinematic transformation just to perform aiming movements regardless of the introduction of a visuomotor rotation [41]. This type of experimental setting has been used in numerous studies on visuomotor adaptation (e.g., [18], [22], [33], [42]–[45]). Conversely, many classic studies of visuomotor adaptation and some more recent ones have used the same plane for visual feedback and hand movements (e.g., [45]–[51]).

Four targets (black circles, 6 mm in diameter) were placed on an invisible circle with a radius of 100 mm around the SP. The directions of the targets were 45°, 135°, 225°, and 315°. Note that 3 o'clock position was 0° and the counterclockwise (CCW) direction had a positive sign. The resulting visual angle between the SP and target positions was 8.61°.

### Procedure

Participants' task was to move the hand from the SP to the target as quickly and precisely as possible. At the beginning of each trial, participants were guided to the SP by one or two out of four red arrows pointing to the left, right, up, and down, appearing in the margins of the monitor. In order to assist the participants in reaching the SP accurately, the SP and the feedback cursor became visible when the stylus was within a radius of 15 mm from the SP. After the cursor had stayed at SP for 500 ms, an auditory signal was delivered to indicate the start of a trial. After a random interval between 500 to 1300 ms, one of the four targets appeared and the SP disappeared. In response, the participants initiated a reaching movement to the target. The completion of the movement was automatically detected by computer software when the cursor was within a tolerance range of 2.7 mm around the target location and the distance between successively sampled positions of the stylus remained equal to or less than 0.25 mm for 400 ms. Then the target and the feedback cursor disappeared, and the trial ended. If the movement lasted longer than 5 s, a beep sound was delivered to notify the participant that the movement was too slow, and he/she was encouraged to move faster in the following trials.

**Figure 1:**
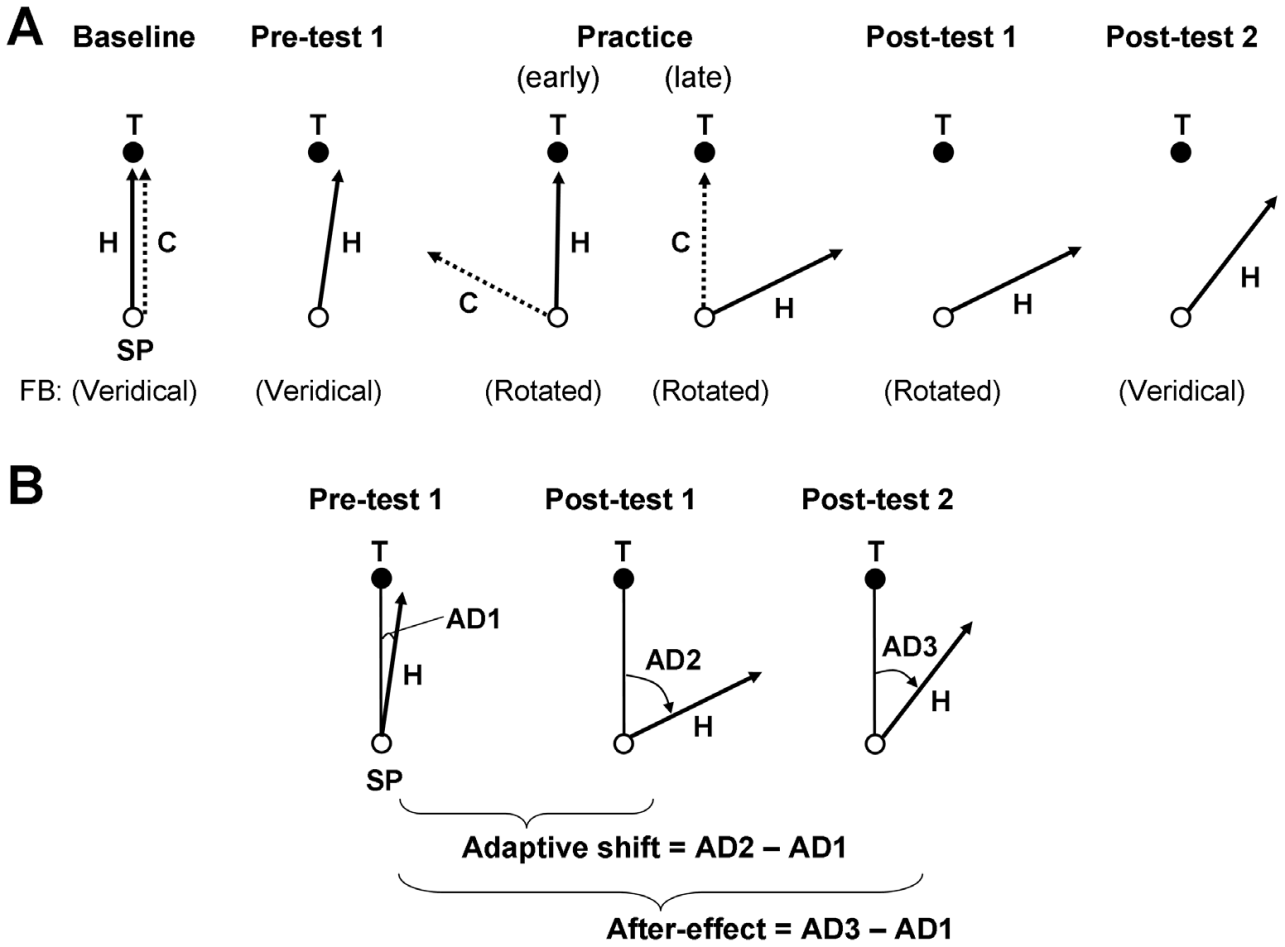
A: Schematic illustrations of selected experimental conditions. Hand movement (H), the starting position (SP) and the target (T) are shown for each condition. Visual feedback (FB) of the hand movement (C) is displayed in the baseline and practice conditions, but not in the pre-test 1, post-test 1 and post-test 2 conditions. Tasks are performed in a veridical-feedback environment for the baseline, pre-test 1 and post-test 2 and in a rotated-feedback environment for the practice and post-test 1. Participants are informed of the type of feedback environment at the beginning of each condition. B: An illustration of calculating adaptive shift and after-effect based on the pre-test 1 and post-tests 1 & 2. The angular deviation (AD) is calculated from the SP-T line to the direction of the hand movement. Adaptive shift (or after-effect) is calculated by subtracting AD1 from AD2 (or AD3). See more details in the text.

The experiment consisted of ten conditions in a fixed order: (1) baseline, (2) pre-test 1, (3) maintenance 1, (4) pre-test 2 (explicit test), (5) practice, (6) post-test 1, (7) maintenance 2, (8) post-test 2, (9) maintenance 3, and (10) post-test 3 (explicit test). These fixed-order conditions were similar to those used in previous studies [31], [52]. Table 1 shows the details of all conditions including the number of trials recorded and analyzed. Schematic illustrations of selected conditions are shown in Figure 1A. On-line visual feedback was displayed on the monitor during the hand movement for conditions 1, 3, 5, 7 and 9, whereas it was not displayed for conditions 2, 4, 6, 8, and 10. For the conditions 2, 6, and 8, the feedback cursor disappeared simultaneously with the appearance of the target. Visual feedback was veridical for conditions 1, 2, 3, 4, and 8, while it was rotated 30°, 75° or 150° in the CCW direction depending on experimental groups for conditions 5, 6, 7, 9, and 10. The participants were informed of the type of visual feedback by the examiner at the beginning of each condition. For all conditions with veridical feedback, participants were informed that the reaching task was easy, but no information about the nature of visual feedback (i.e., veridical) was provided. For all conditions with rotated visual feedback, participants were informed that the reaching task was more difficult than the easy one, but no information about the introduction of a visuomotor rotation was provided. For the conditions where the display of visual feedback was absent, participants were instructed to perform the task as if it were an easy one (i.e., veridical visual feedback for conditions 2, 4, and 8) or a more difficult one (i.e., rotated visual feedback for conditions 6 and 10), even though no visual feedback would be displayed on the monitor.

**Table 1:** Experimental conditions.

	Condition	Number of trials	Display of visual feedback	Type of visual feedbackb)
1	baseline	12a)	present	veridical
2	pre-test 1	14	absent	veridical^c)^
3	maintenance 1	8	present	veridical
4	pre-test 2 (explicit test)	14	absent	veridical^c)^
5	practice	160	present	rotated
6	post-test 1	14	absent	rotated^d)^
7	maintenance 2	8	present	rotated
8	post-test 2	14	absent	veridical^c)^
9	maintenance 3	8	present	rotated
10	post-test 3 (explicit test)	14	absent	rotated^d)^

a) : First four trials were warm-up trials and not analyzed.

b) : Participants were informed of the type of visual feedback by the examiner at the beginning of each condition.

c), d) : For the conditions where the display of visual feedback was absent, participants were instructed to perform the task as if it were in a veridical-feedback environment (^c^) or in a rotated-feedback environment (^d^). See more details in the text.

During the baseline condition, participants familiarized themselves with task procedures of reaching movements to a target and receiving visual feedback, and the last eight trials of this condition was used to examine the baseline performance. Pre-test 1 was used to examine the baseline performance under no on-line visual feedback of the hand movement. Pre-test 2 was an explicit test, which examined the baseline level of participants' explicit knowledge of aiming direction to the target. For this purpose, the SP and the target were presented together with a line starting from the SP. The length of the line was slightly shorter than the SP-target distance. The line was initially presented at an angle of 150° clockwise (CW) from the SP-target direction, and it slowly moved around the SP. Participants instructed the experimenter to stop and finely adjust the orientation of the line so that it matched the reaching direction to the target under the absence of visuomotor rotation. Between the pre-tests 1 and 2, participants performed maintenance 1 condition, which was identical to the baseline condition. The pre-test 2 was followed by practice condition with a visuomotor rotation (Table 1).

After the practice condition, the participants underwent 3 types of post-tests (1, 2, and 3, see Table 1) to assess different types of knowledge of the visuomotor rotation acquired through practice [20], [41], [43]. Performance without on-line visual feedback of the movement was first tested under the presence of the visuomotor rotation as post-test 1. This test differed from the pre-test 1 only with respect to the introduction of a visuomotor rotation, of which the participants were informed, even though they did not see the feedback of their hand movements on the monitor. The changes of movement direction from the pre-test 1 to the post-test 1 were measured as adaptive shifts (Fig. 1B), which reflected both implicit adjustments and explicit strategic adjustments of hand directions. Adaptive shifts did not reflect behavioral changes due to on-line corrections of movements, because both pre-test 1 and post-test 1 were performed without on-line feedback.

Post-test 2 was the same as pre-test 1 and differed from the post-test 1 with respect to the absence of any rotation. The participants did not see the feedback of their hand movements on the monitor, but were informed that the visuomotor rotation was no longer present. For this reason, there was no reason for the participants to apply explicit strategic adjustments. Thus, any changes of movement direction from the pre-test 1 to the post-test 2 (measured as after-effects, Fig. 1B) reflected only implicit adjustments of hand directions acquired through practice. Similar to the adaptive shifts, after-effects did not reflect behavioral changes due to on-line corrections of movements, because both pre-test 1 and post-test 2 were performed without on-line feedback.

Post-test 3 was identical with pre-test 2 (explicit test) except that participants judged the direction of the line that matched the reaching direction for bringing the cursor to the target under the presence of visuomotor rotation. Since the participants were informed of the presence of a visuomotor rotation in post-test 3, any changes of judgments of movement direction from pre-test 2 (where participants were informed of the absence of the rotation) reflected explicit knowledge of the rotation acquired through practice. These changes were measured as explicit shifts. Between the post-tests, participants performed maintenance 2 and 3 conditions, respectively, which were identical to the practice condition.

For hand movements, the x- and y-positions of the tip of the stylus were recorded by the digitizer at 133 Hz with a spatial resolution of 0.005 mm. The x- and y-positions of eye movements were recorded at 500 Hz by the eye tracker. For each of x- and y-positions, spatial resolution of the eye tracking system was 0.03° and its accuracy was 0.4°. Left and right eye positions were averaged by the time of recording. The eye tracker was calibrated for each participant before data recording by using nine calibration points displayed across the monitor. For synchronization of eye and hand data recording, data acquisition software written in C++ simultaneously initiated the recording of the digitizer and eye tracker.

### Data analysis

#### Analysis of hand movements

The x- and y-positions of the stylus were resampled at 500 Hz, filtered with a Butterworth filter (low-pass, 4^th^ order, 25 Hz cutoff frequency), and differentiated by using a two-point central-difference algorithm to obtain velocity. Hand movement onset and offset were defined as follows. Starting from the peak tangential velocity, data points where tangential velocity was less than 5 mm/s the first time and remained smaller for 200 ms thereafter were searched in both the forward (for the movement offset) and backward (for the movement onset) directions. These points were determined as onset and offset [31]. The results of this automatic procedure were inspected and corrected manually as needed.

For each trial of the baseline and practice conditions, movement time, trajectory length and initial direction error were computed. Hand movement time was measured from movement onset to offset. Hand trajectory length was measured as the cumulative resultant distance of the hand path from movement onset to offset. Note that the value of this measurement increased if the hand path was not straight. For the analysis of initial direction errors, a reference line was defined as the line connecting the hand position at movement onset and the center of the target (in the case of baseline condition) or the hand position that would place the cursor on the target center under the rotated feedback (in the case of practice condition). The initial direction was computed as the angular deviation from the reference line to the line connecting the hand positions at movement onset and at 10 mm distance point from the onset (see the use of a similar procedure [53]). The initial direction was positive (or negative) when a hand-path was directed CCW (or CW) to the reference line.

Adaptive shift, after-effect, and explicit shift were measured by comparing results of pre-tests and post-tests. For each trial of pre-test 1 and post-tests 1 and 2, terminal direction error was computed as the angular deviation of the line connecting the center of SP and hand position at the movement completion from the line connecting the centers of SP and the target (the SP-target line, Fig. 1B). Subsequently, the difference between mean terminal direction error across the first four trials of post-test 1 (or post-test 2) and that across the first four trials of pre-test 1 was calculated as adaptive shift (or after-effect). For each trial of explicit tests, a judged rotation angle was computed as the angular deviation of the judged direction of the line by the participants from the direction of the SP-target line. Subsequently, explicit shift was measured as the difference between mean judged rotation angle across the first four trials of pre-test 2 and that across the first four trials of post-test 3.

#### Analysis of eye movements

**Figure 2:**
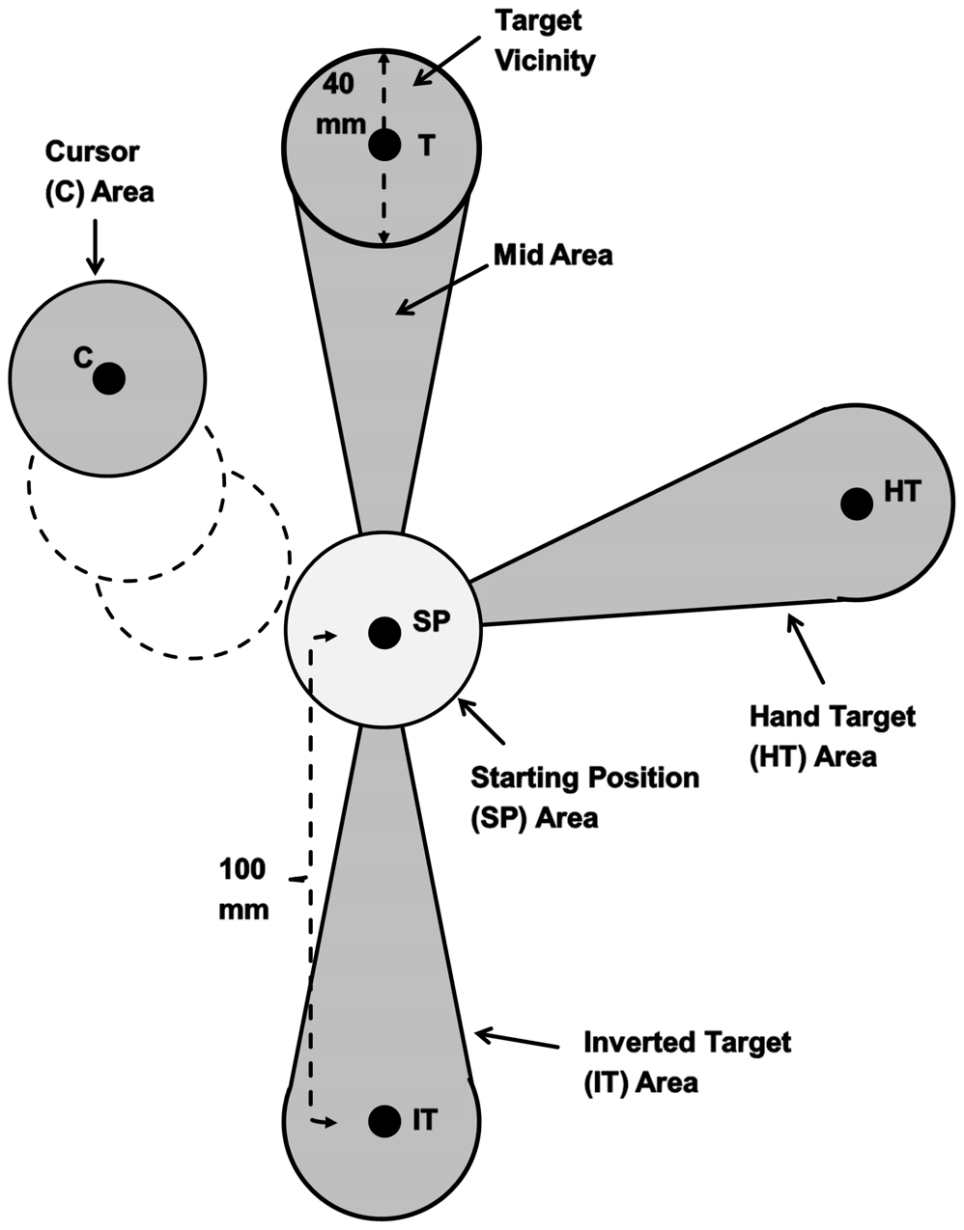
Different gaze areas used for analysis of gaze locations. Five black dots indicate the starting position (SP), the target (T), the hand target (HT), the inverted target (IT), and the cursor (C) positions. The HT position is the hand location that would bring the cursor to the target under the rotated visual feedback (example shown for the 75° rotation). The IT position is the 180° inverted location of the target. The starting position (SP) area, the cursor (C) area, and the target vicinity have a circular shape. The mid area is an area between the SP and target vicinity. The hand-target (HT) area and the inverted-target (IT) area have the same shape as the combined area of the target vicinity and the mid area.

Analyses of eye movements were carried out based on unfiltered x- and y- position data to avoid any types of distortion of signal through filtering. Missing data points were linearly interpolated. However, these points were not included in the present results. To investigate when gaze anchoring to the target occurred during hand movement, each trial of the baseline and practice conditions were analyzed in the following steps. First, target vicinity was defined as a 20 mm radius around the center of the target position (Fig. 2). The visual angle of target vicinity was 3.47°. Second, the latest time at which gaze entered this area and remained there until hand movement offset was identified as the onset of gaze anchoring. If there was a very short exit of gaze from this area during gaze anchoring (<50 ms), it was considered as spurious and was not registered as the latest time. This landmark was automatically detected by using computer software. Subsequently, the results of this automatic procedure were inspected and corrected manually as needed. Third, a period from the hand movement onset to the latest time was defined as the pre-gaze anchoring (pre-GA) period, and its duration was measured as pre-GA duration. Fourth, resultant trajectory length during that period (pre-GA trajectory length) was measured and expressed as the percentage of total trajectory length. In the case of trials where gazes were already in the target vicinity at hand movement onset, the values of the pre-GA period and trajectory length were recorded as zero.

To further explore gaze locations prior to gaze anchoring to the target, different gaze areas were defined within the working space (Fig. 2). The cursor area was a circle (20 mm radius) centered on the feedback cursor. The starting position area (SP area) was a circle (20 mm radius) centered on SP. The mid area was an area between the SP area and the target vicinity. The mid area (Fig. 2) was defined by two lines, each of which was originating from the center of SP and ending at each outer edge of target vicinity; it did not include any overlap with the SP area or the target vicinity. We defined this area empirically based on our observation of a large sample of eye position data as well as an observation by Sailer et al. where gazes tended to fall in an area between the start point and the goal during learning [17]. The size of foveal vision is known to be about 2° in diameter [54]–[57]; and hence, if a gaze moved from the SP to the target in a straight path, the mid area mostly covered the area of foveal vision along that path.

The hand target area and the inverted target area were defined in a similar manner as the combined area of the target vicinity and the mid area (see Fig. 2). The hand target area was related to the hand position (HT, Fig. 2) that would bring the cursor to the target center under the rotated visual feedback. The inverted target area was related to the position (IT, Fig. 2) that would be obtained by a 180° polarity inversion of both axes of the target center. The remaining area in the working space outside the six gaze areas (cursor, SP, mid, hand target, and inverted target areas as well as target vicinity) was defined as “the other area”. For the gaze location analysis, the total duration of gaze falling in each area as well as each of two overlapped areas (between the cursor and SP areas and between the cursor and mid areas) was measured during the pre-GA period and expressed as the percentage of the pre-GA duration.

Additionally, the average distance between the center of the feedback cursor and gaze location was calculated during the pre-GA period to examine if gazes shifted away from the feedback cursor over the course of practice. The distance between the cursor center and gaze location was calculated based on x- and y-positions in each data frame. Subsequently, the average distance across all sampling frames in the pre-GA period was calculated for each participant.

Trials with bad hand recording (e.g., started the movement before target presentation, the hand went out of recording range) or bad eye recording (e.g., blinking, missing data points precluded a reliable determination of gaze entrance to the target vicinity) were eliminated from the further analysis (165 trials: 2.31% of all recorded trials except those from explicit tests). The eye data were screened for outliers among participants in terms of baseline performance of gaze anchoring behavior. Based on the pre-GA trajectory length in the baseline condition, a mean and a standard deviation across all participants of all groups were calculated, and the values outside the range of mean ±2.5SD were defined as outliers. As a result, two participants in the 30° group were identified as having outliers, and were excluded from all analyses. For analysis of pre-tests and post-tests, the data were screened for outliers among all trials within each test of each participant. Trials with values outside the range of mean ±2.5SD were eliminated as outliers. As a result, 9 trials (1.61%) out of 560 trials (first four trials of all pre-tests and post-tests) were removed from the analysis. For analysis of gaze locations during the pre-GA period, artifacts of eye data were removed based on visual inspection. Due to this removal and the exclusion of interpolated data points, 1.26% of the pre-GA duration average across all trials was not analyzed.

### Statistical analysis

For the baseline condition, a mean value across the last eight trials was calculated in each participant. For the practice condition, all trials were divided into 40 blocks with four trials each. A mean value was calculated across trials for each block, and this value was used to calculate the group's mean for that block. Group difference in baseline performance was tested by using a one-way ANOVA. To assess changes of hand or eye movements from the beginning to the end of the practice condition, a mean value of the first three blocks (i.e., early practice phase) and that of the last three blocks (i.e., late practice phase) were calculated for each participant in each group. These values were compared by using a 3×2 mixed design ANOVA with group as a between-subject factor (30°, 75° and 150°) and phase as a within-subject factor (early and late practice). When group-by-phase interaction was significant, a difference score between the early and late practice phases was calculated in each participant, and this value was subjected to post-hoc analysis. Post-hoc comparisons were performed using Bonferroni corrected t-tests. Furthermore, to assess whether movements at the end of the practice condition differed from baseline performance, the mean value in the baseline condition and that of the late practice phase were compared by using a paired t-test in each group.

For three parameters regarding pre- and post-tests (i.e., adaptive shift, after-effect, and explicit shift), all statistical analyses were carried out by using nonparametric tests because all three parameters of the 150° group violated normality of the distribution. Group difference was examined in each pair among the three groups by using a Bonferroni corrected two-sample Kolmogorov-Smirnov test. The one-sample Wilcoxon signed-rank test was used to test whether the after-effects and explicit shifts were different from zero degrees (or 180° only in the case of explicit shift for the 150° group). The probability level for statistical significance was *p*<0.05.

## Results

We will first report adaptive changes of hand movements and results of post-tests, followed by adaptive changes of eye movements.

### Hand movements

#### Movement time

**Figure 3:**
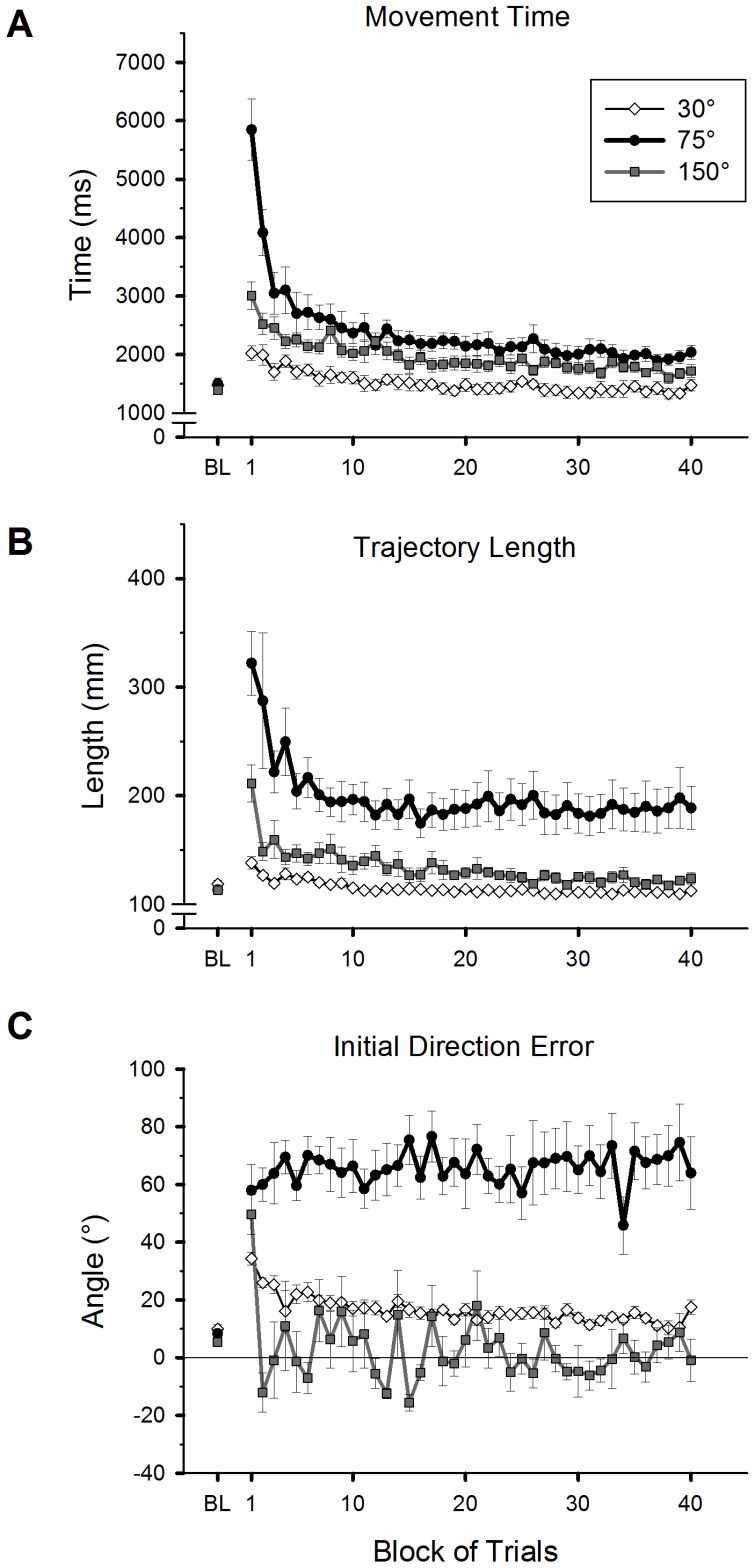
Adaptive changes of movement time (A), total trajectory length (B) and initial direction error (C) for hand movements during practice of a visuomotor rotation. Mean values of all participants are plotted against 40 trial blocks with 4 trials each for the 30° (white diamonds), 75° (black circles), and 150° (grey squares) groups. Mean values from the baseline condition (BL) are also plotted. The error bars represent standard errors.

When no visuomotor rotation was present in the baseline condition, mean movement time across all participants was 1456.3±42.2 [SE] ms (Fig. 3A), and there was no group difference (one-way ANOVA, *p*>0.05). Movement time substantially increased in all groups in the early practice phase after a visuomotor rotation was introduced. The 75° rotation group (Fig. 3A, black circles) made the longest movement time among the groups, followed by the 150° group (Fig. 3A, grey squares). The 30° group made the shortest time (Fig. 3A, white diamonds). Movement time gradually decreased over the course of practice. The observed movement time in this study was within the range of those reported in previous studies that allowed on-line feedback corrections during the movement [28], [29], [31], [49], [58]. A 3 (group: 30°, 75° and 150°)×2 (phase: early and late practice) ANOVA revealed a significant group effect (*F*(2,25) = 27.61, *p*<0.001). The 75° group had significantly longer movement time than the 30° and 150° groups (post-hoc, *p*<0.001 for both comparisons), which did not differ from each other (*p*>0.05). There was a significant phase effect (*F*(1,25) = 94.34, *p*<0.001) and a group-by-phase interaction (*F*(2,25) = 17.08, *p*<0.001). To further examine the interaction, a difference score from the early to the late practice phase was calculated. Mean difference score of the 75° group (2354.5±326.3 ms) was significantly greater than those of the 30° (526.8±84.7 ms) and 150° (996.8±159.0 ms) groups (post-hoc, *p*<0.01 for both comparisons), which did not significantly differ from each other (*p*>0.05).

To examine if the performance at the end of practice was similar to the baseline performance, movement time of the late practice phase was compared to that of the baseline condition in each group. The 30° group did not show any significant difference between the two performances, while the other two groups did (75°: *t*(9) = 6.11, *p*<0.001; 150°: *t*(9) = 4.07, *p*<0.01).

#### Trajectory length

Total trajectory length of hand movements from the starting position to the target (Fig. 3B) exhibited relatively similar adaptive changes as movement time. There was no group difference in the baseline condition (one-way ANOVA, *p*>0.05). When the visuomotor rotation was introduced, the 75° group increased the trajectory length most among the groups, followed by the 150° group. Trajectory length decreased over the course of practice in all groups. The 75° group maintained a much longer trajectory length than the other groups throughout practice.

Confirming these observations, a 3×2 ANOVA revealed a significant group effect (*F*(2,25) = 13.17, *p*<0.001), a phase effect (*F*(1,25) = 30.15, *p*<0.001), and a group-by-phase interaction (*F*(2,25) = 4.29, *p*<0.05). The 75° group produced significantly longer trajectories than the 30° and 150° groups (post-hoc, *p*<0.01 for both comparisons), which did not differ from each other (*p*>0.05). The interaction effect stemmed from a significantly greater difference score between the early and late practice phases for the 75° group (85.3±22.5 [SE] mm) compared to that of the 30° group (16.9±3.3 mm, post-hoc, *p*<0.05). However, the mean difference score of the 150° group (52.1±12.7 mm) did not significantly differ from those of the 30° and 75° groups (*p*>0.05 for both comparisons). When the performances of the late practice phase and the baseline condition were compared, the 75° and 150° groups still showed significant differences (75°: *t*(9) = 3.71, *p*<0.01; 150°: *t*(9) = 4.00, *p*<0.01), whereas the 30° group did not (*p*>0.05).

#### Initial direction error

Initial direction error of hand movements was measured to assess if participants changed preplanned reaching direction through practice (Fig. 3C). There was no group difference in the baseline condition (one-way ANOVA, *p*>0.05). Immediately after the introduction of the visuomotor rotation, all three groups increased the initial direction error. The 30° group gradually reduced the error, while the 75° group (Fig. 3C, black circles) maintained a large direction error throughout practice. The 150° group made a positive direction error in the first practice block (Fig. 3C, grey squares). But they made a negative error (−12°) in the next block, suggesting that participants began to introduce a reversal shift for hand movements (note that a 180° inversion would produce a −30° error). Thereafter, the initial direction error substantially fluctuated between ±20° around 0° for the rest of practice. The mean value in the late practice phase was 4.3°.

A 3×2 ANOVA revealed a significant group effect (*F*(2,25) = 33.62, *p*<0.001). The 75° group produced significantly greater errors than the other two groups (post-hoc, *p*<0.001 for both comparisons), which did not differ from each other (*p*>0.05). Although the phase effect was not significant (*p*>0.05), a group-by-phase interaction was significant (*F*(2,25) = 3.86, *p*<0.05). The interaction effect stemmed from a significantly greater difference score between the early and late practice phases for the 30° group (15.7±2.1 [SE] mm) compared to that of the 75° group (−8.9±8.7 mm, post-hoc, *p*<0.05). However, the mean difference score of the 150° group (7.8±5.2 mm) did not significantly differ from those of the 30° and 75° groups (*p*>0.05 for both comparisons). The initial direction error in the late phase (LP) was significantly different compared to that of the baseline (BL) for the 30° (LP: 12.8° [mean], BL: 9.9°, *t*(7) = 2.98, *p*<0.05) and 75° groups (LP: 69.5°, BL: 8.3°, *t*(9) = 5.37, *p*<0.001), and there was no difference in the 150° group (LP: 4.4°, BL: 5.4°, *p*>0.05).

It should be noted that the unchanged initial direction error found in the 75° group was different from other studies that showed a reduction of this parameter during practice of 60°–90° visuomotor rotations [21], [31], [42], [59]. This difference was caused by a subgroup of the participants increasing the direction error during practice, while the other subgroup decreased it (see Appendix 1 in Text S1 and Fig. S1A). A further analysis of the curvature of hand paths has revealed that the former subgroup produced greater curvature than did the latter, which nonetheless produced greater curvature than the 30° and 150° groups (Appendix 1 – Fig. S1B).

In summary, both movement time and trajectory length were reduced during practice in all three groups. Initial direction error was also reduced for the 30° and 150° rotation groups, whereas no reduction was found in the 75° group. At the end of practice, only the 30° group was able to restore a performance similar to the baseline.

#### Adaptive shifts

**Figure 4:**
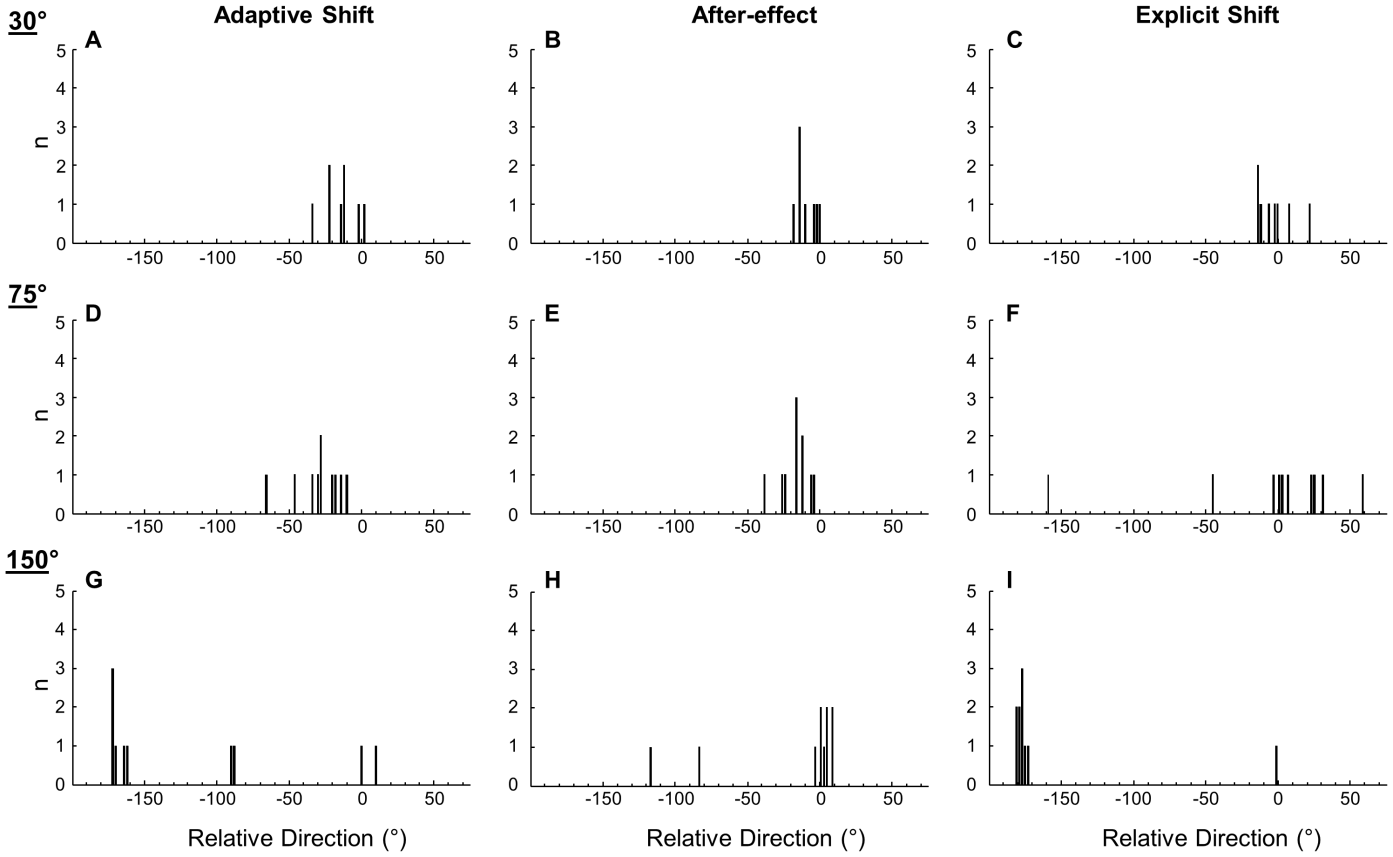
Adaptive shifts, after-effects and explicit shifts. Histograms of the adaptive shits (left column), after-effects (center column), and explicit shifts (right column) are plotted for the 30° (A-C), 75° (D-F), and 150° (G-I) groups. The binning size of each plot is 2°.

Three parameters (adaptive shift, after-effect, and explicit shift) were measured to assess different types of knowledge of the visuomotor rotation acquired through practice by comparing results of pre-tests and post-tests [20], [41], [43]. Adaptive shift measured the magnitude of adaptive changes due to practice, which excluded changes attributed to the on-line visual feedback control. This parameter is thought to reflect implicit corrections based on implicit knowledge of the visuomotor rotation and strategic corrections based on explicit knowledge of the rotation. Adaptive shifts of −30°, −75°, and −150° would completely compensate for the visuomotor rotations of 30°, 75°, and 150°, respectively. Histograms (with the binning size of 2°) of adaptive shifts for all three groups are shown in Fig. 4 (left panels). The group median values were −13.1° for the 30° group (Fig. 4A) and −27.3° for the 75° group (Fig. 4D). Thus, both groups produced smaller adaptive shifts compared to the magnitudes of respective visuomotor rotations. In contrast, the 150° group made either smaller or greater adaptive shifts compared to the magnitude of the visuomotor rotation (Fig. 4G). The median value was −163.3°. The 150° group had significantly greater adaptive shifts than the other two groups (30°: *D*(18) = 0.800, *p*<0.05; 75°: *D*(20) = 0.800, *p*<0.01), which did not differ from each other (*p*>0.05).

#### After-effects

After-effect measured the magnitude of implicit knowledge of the visuomotor rotation acquired through practice. After-effects of −30°, −75°, and −150° would indicate that participants acquired perfect implicit knowledge of the visuomotor rotations for 30°, 75°, and 150°, respectively. Histograms (with the binning size of 2°) of after-effects for all three groups are shown in Fig. 4 (center panels). The 30° and 75° groups produced similar after-effects (median values: −11.4° and −15.5°, respectively, Fig. 4B, E). For the 150° group, however, the majority of participants produced little after-effects, and the median value was 2.6° (Fig. 4H). The difference between 150° and 75° groups was significant (*D*(20) = 0.800, *p*<0.01). The 30° group did not differ from the other groups (*p*>0.05 for both comparisons). Furthermore, the one-sample Wilcoxon signed rank test applied in each group revealed that the observed after-effects were significantly greater than 0° for the 30° group (*T*(8) = 2.00, *p*<0.05) and the 75° group (*T*(10) = 0.00, *p*<0.01), while those of the 150° group were not (*p*>0.05). This indicates that implicit knowledge of the visuomotor rotation was present only for the 30° and 75° groups.

#### Explicit shifts

Explicit shift measured the magnitude of explicit knowledge of the visuomotor rotation acquired from practice. The values of −30°, −75°, and −150° would indicate that participants acquired perfect explicit knowledge of the visuomotor rotations for 30°, 75°, and 150°, respectively. Histograms of explicit shifts for all three groups are shown in Fig. 4 (right panels, binning size: 2° for each plot). The 30° group made relatively small explicit shifts (median value: −2.8°, Fig. 4C). The 75° group had the median value of 5.0°, but there was large inter-individual variability (SE: 19.2°, Fig. 4F). Conversely, the 150° group had small inter-individual variability (SE: 1.0°) except for one participant (Fig. 4I). This group's median value was −177.1°. The 150° group made significantly greater explicit shifts than the other two groups (30°: *D*(18) = 0.900, *p*<0.01; 75°: *D*(20) = 0.900, *p*<0.01), which did not differ from each other (*p*>0.05). In addition, the observed explicit shifts were not significantly greater than 0° for the 30° and 75° groups (*p*>0.05 in each group), indicating that there was no explicit knowledge of the visuomotor rotation. For the 150° group, the explicit shifts were significantly smaller than 180° (*T*(10) = 0.00, *p*<0.01), indicating that the explicit knowledge was slightly but significantly smaller than the value that would reflect the inverted target location for 180°.

In summary, the 30° group acquired some implicit knowledge (about two fifth) of the applied rotation, and so did the 75° group (about one fifth). However, neither group acquired explicit knowledge of the applied rotation. The 150° group acquired explicit knowledge as if it were a 180° rotation, but no implicit knowledge.

### Eye movements

#### Gaze anchoring during hand movement

**Figure 5:**
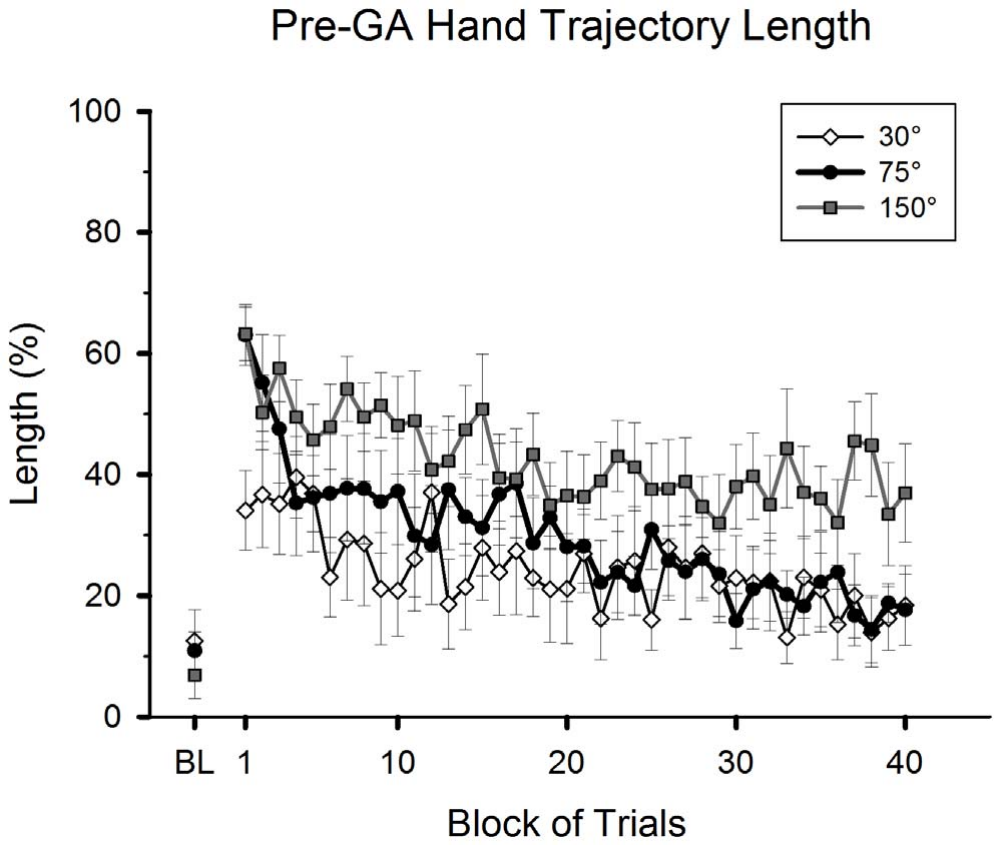
Adaptive changes of gaze anchoring behavior. Hand trajectory length in the pre-gaze anchoring (Pre-GA) period is plotted against block of trials during practice of a visuomotor rotation. The values are expressed as a percentage of the total trajectory length. Formats of the plot are the same as in Fig. 3. The error bars represent standard errors.

To examine whether the gaze anchoring behavior to the target was altered over the course of practice, the last timing of gaze entrance to the target vicinity during hand movement was identified in each trial. The period from the onset of hand movement to the last timing was defined as the pre-gaze anchoring (pre-GA) period. Subsequently, a trajectory length of hand movement during the pre-GA period was measured and expressed as the percentage of total hand trajectory length (Fig. 5). Group mean values of the pre-GA trajectory length ranged from 7% to 13% in the baseline condition, showing that gaze anchoring to the target vicinity occurred from the very early phase of hand movements. There was no group difference (one-way ANOVA, *p*>0.05).

Immediately after the visuomotor rotation was introduced, mean pre-GA trajectory lengths increased substantially in all groups (Fig. 5), showing that the timing of the gaze anchoring to the target vicinity was much delayed during the hand movement. This change was more pronounced for the 75° and 150° groups compared to the 30° group. All groups gradually reduced the trajectory lengths throughout practice, indicating that the timing of gaze anchoring was shifted to the earlier phase of hand movement. The rate of the reduction in the 75° group (Fig. 5, black circles) was steeper than those in the 30° and 150° groups. At the late practice phase, only the 30° and 75° groups reached a timing of gaze anchoring similar to the baseline performance.

In addition, the subgroup of participants in the 75° group who decreased the initial direction error of hand movements during practice (Appendix 1 – Fig. S1C, black circles) showed a relatively similar change of gaze anchoring behavior as that of the 30° group (Fig. 5, white diamonds). This subgroup more rapidly decreased the pre-GA trajectory length than did the other subgroup, which increased the initial direction error of hand movements during practice (Appendix 1 – Fig. S1C, white circles).

A 3 (group: 30°, 75° and 150°)×2 (phase: early and late practice) ANOVA revealed that all main effects were significant (group: *F*(2,25) = 4.50, *p*<0.05; phase: *F*(1,25) = 51.57, *p*<0.001; group-by-phase interaction: *F*(2,25) = 3.54, *p*<0.05). There was a significant difference between the 30° and 150° groups (post-hoc, *p*<0.05), but no other group difference was found. The interaction effect was attributed to that the mean difference score from the early to late practice phase for the 75° group (38.3±5.5 [SE] %) tended to be greater than that for the 150° group (18.6±7.5%, post-hoc, *p* = 0.076). However, the mean difference score of the 30° group (19.1±3.7%) did not differ from those of the 75° and 150° groups (*p*>0.05 for both comparisons). When the pre-GA trajectory lengths at the end of practice was compared with those of the baseline condition, the 30° and 75° groups did not show any significant difference (t-test, *p*>0.05 in each group), whereas the 150° group did (*t*(9) = 4.39, *p*<0.05, Fig. 5).

In summary, all groups delayed the onset of gaze anchoring to the target in the early practice phase. This delay was smallest for the 30° group. All groups gradually advanced that onset throughout practice. However, only the 30° and 75° groups restored onset timing similar to the baseline performance by the end of practice.

#### Gaze locations prior to gaze anchoring

**Figure 6:**
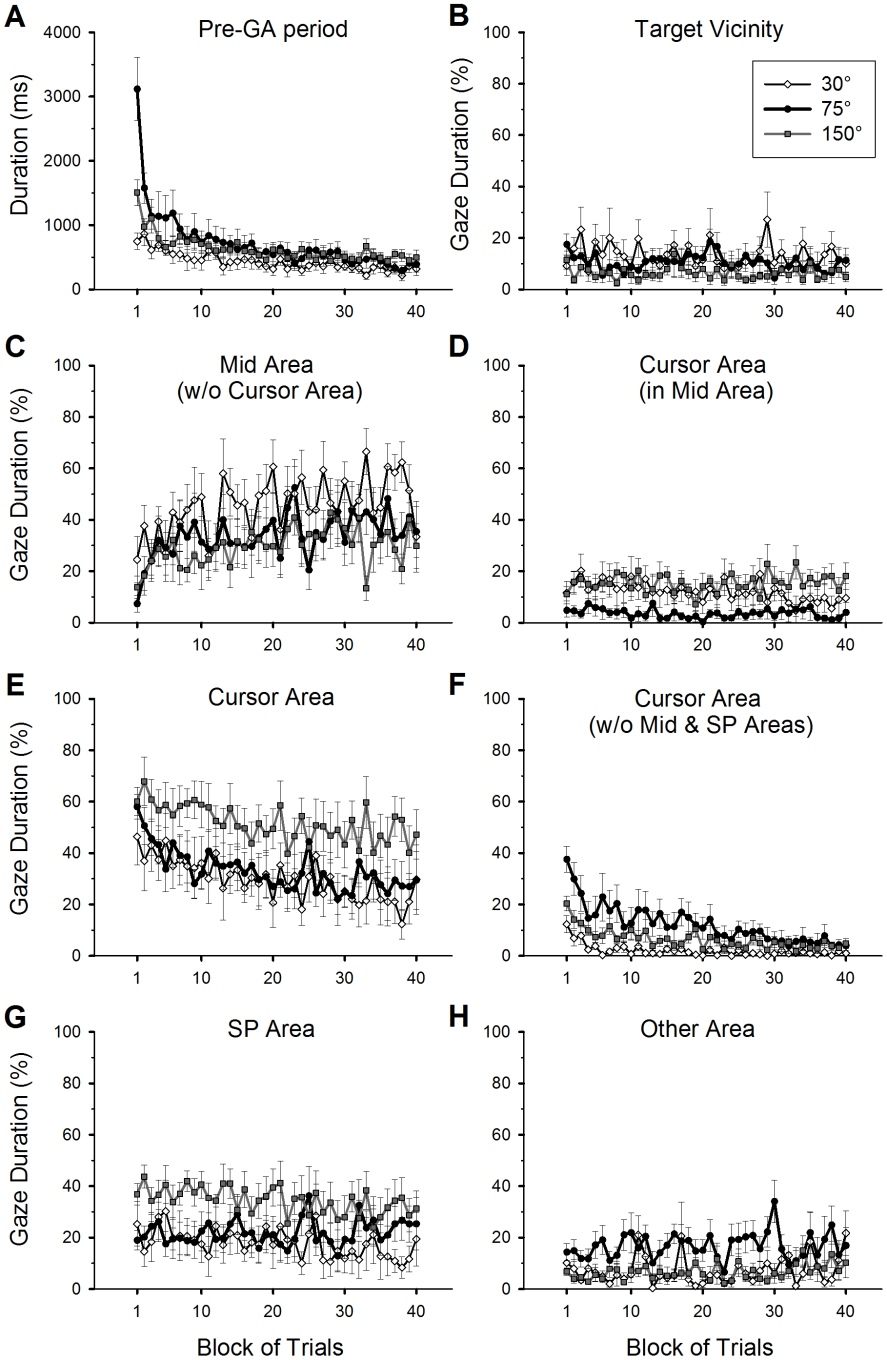
Adaptive changes of hand movement time (A) and gaze locations (B-H) in the pre-gaze anchoring (Pre-AG) period during practice of a visuomotor rotation. Gaze durations are plotted for different areas of work space (Fig. 2): the target vicinity (B), the mid area excluding (w/o) an overlap with the cursor area (C), the cursor area within the mid area (D), the cursor area (E), the cursor area excluding overlaps with the mid and starting position (SP) area (F), and other area (H). The other area is the work space outside the target vicinity, mid, cursor, SP, hand-target, and inverted-target areas (see Fig. 2 for details). Gaze duration is expressed as the percentage of the pre-GA period. Formats of the plots are the same as in the part related to the practice condition of Fig. 3. The error bars represent standard errors.

Adaptive changes of the pre-GA duration and gaze locations are shown in Fig. 6. Note that trials where gaze was already in the target vicinity at the hand movement onset were not included for these analyses. In this section, results of post-hoc analysis of a group effect are reported only for those with a significant difference in order to make a succinct data presentation. Duration of the pre-GA period is shown in Fig. 6A. At the early practice phase, the 75° group produced the longest duration, followed by the 150° group, whereas the 30° group had the shortest duration. All groups gradually shortened the duration during practice, and the group difference was minimal at the end of practice. A 3×2 ANOVA revealed all significant main effects (group: *F*(2,25) = 5.89, *p*<0.01; phase: *F*(1,25) = 79.54, *p*<0.001; group-by-phase interaction: *F*(2,25) = 11.32, *p*<0.001). The pre-GA duration was significantly longer for the 75° group compared to the 30° group (post-hoc: *p*<0.01). The interaction was attributed to a significantly greater difference score between the early and late phases for the 75° group (1573.2±228.3 [SE] ms) compared to those of the other two groups (30°: 437.5±124.3 ms; 150°: 717.5±135.1 ms, *p*<0.01 for both comparisons), which did not differ from each other (*p*>0.05).

To examine gaze locations during the pre-GA period, total duration of gazes falling in each of various gaze areas (Fig. 2) was measured and expressed as the percentage of the pre-GA duration. All groups showed relatively low percentages of looking at the target vicinity (Fig. 6B). No significant main effect was found (*p*>0.05).

Regarding gazing at the mid area between the starting position (SP) area and target vicinity (Fig. 6C), we first measured the gaze duration by excluding gazes that fell in the cursor area within the mid area. This enabled us to separate gazes that were falling in this area from those that were following the feedback cursor. Gaze duration for the mid area significantly increased from the early to the late practice phase (*F*(1,25) = 14.04, *p*<0.01). There was no group effect or group-by-phase interaction (*p*>0.05). Next, we analyzed the duration of gazes that fell in only the cursor area within the mid area (Fig. 6D). All groups showed relatively short durations. However, the difference between the 75° and 150° groups was significant (group effect: *F*(2,25) = 4.39, *p*<0.05; post-hoc, *p*<0.05). There was no other main effect (*p*>0.05).

Regarding gazing at the cursor area that included the overlaps with the SP and mid areas (Fig. 6E), the 75° and 150° groups looked at the cursor area in the majority of the pre-GA period at the beginning of practice. Conversely, the 30° group looked at it less than did the other groups. All groups decreased the duration of cursor gazing throughout practice. The 150° group maintained long duration until the end of practice. To confirm these observations, there were significant group (*F*(2,25) = 4.44, *p*<0.05) and phase effects (*F*(1,25) = 15.94, *p*<0.001). The difference between the 150° and 30° group was significant (post-hoc, *p*<0.05). There was no group-by-phase interaction (*p*>0.05).

For the cursor area excluding overlaps with the SP and the mid areas (Fig. 6F), the 75° group spent more time to gaze at the cursor area than did the other two groups in the initial half of the practice condition. All groups gradually decreased the gaze duration for the cursor area throughout practice, and the group difference was minimal at the end of practice. There was a significant phase effect (*F*(1,25) = 58.66, *p*<0.001), a group effect (*F*(2,25) = 7.83, *p*<0.01), and a group-by-phase interaction (*F*(2,25) = 7.87, *p*<0.01). The 75° group gazed at the cursor area significantly more than did the 30° group (post-hoc: *p*<0.01). The interaction stemmed from a significantly greater difference score between the early and late practice phases in the 75° group (26.6±4.6 [SE] %) compared to those of the 30° (8.1±2.1%) and 150° groups (12.0±2.7%, post-hoc, *p*<0.05 for both comparisons), whereas the latter two groups did not differ from each other (*p*>0.05).

**Figure 7:**
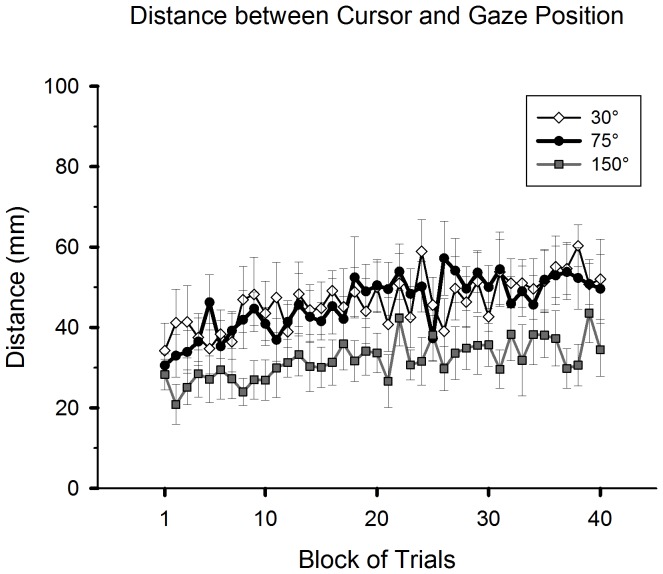
Adaptive changes of average distance between the feedback cursor and gaze location during the pre-gaze anchoring (Pre-GA) period. The format of the plot is the same as in the part related to the practice condition of Fig. 3. The error bars represent standard errors.

The above results indicate that gazes shifted away from the feedback cursor over the course of practice. This was confirmed by a gradual increase of the average distance between the cursor and gaze location during practice in all groups (Fig. 7), and the phase effect was significant (*F*(1,25) = 23.29, *p*<0.001). The 150° group had shorter distance between the cursor and gaze location than the other groups, confirming the result shown in Fig. 6E. There was a significant group effect (*F*(2,25) = 4.44, *p*<0.05), and the difference between the 150° and 30° groups was significant (post-hoc, *p*<0.05). There was no group-by-phase interaction.

Gazes that fell in the SP area (Fig. 6G) mostly overlapped with those in the cursor area within the SP area. The proportion of such overlap relative to the overall SP-area gaze duration shown in Fig. 6G was 83.5% (30° group), 75.6% (75° group), and 81.5% (150° group) across all trial blocks. The 150° group spent more time to gaze at the SP area than did the other two groups. The group effect was significant (*F*(2,25) = 6.76, *p*<0.01), and the 150° group significantly differed from the 30° group (post-hoc: *p*<0.01). There was no other main effect (*p*>0.05). In addition, the percentages of looking at the hand-target area and the inverted-target area (Fig. 2) were negligible for all groups throughout the practice condition (mean value across 40 trial blocks was less than 0.6% for each area). The percentages of gazing at areas outside the above areas (target vicinity, mid, cursor, SP, hand-target, and inverted-target areas) were relatively small (Fig. 6H). No main effect was found in this area (*p*>0.05).

In summary, during the pre-gaze anchoring period, all groups gradually shifted their gaze patterns from looking at the vicinity of the cursor to looking at the mid area between the SP and the target. The 150° group looked significantly more at the cursor area than did the other groups. The 75° group gazed at the cursor area for a longer time only in the relatively early period of practice.

## Discussion

### Adaptive changes of hand movements

We examined adaptive changes of hand movements and eye-hand coordination during a visuomotor rotation task with different rotation angles (30°, 75°, and 150°). Regarding hand movements of the 30° group, the observed adaptive shift (−13°) was similar to that of the previous study [31] but fell short of the value (−30°) that would fully compensate for the 30° rotation. The difference between these values reflects rotational compensation attributed to the feedback control [41]. At the same time, the participants adjusted preplanned directions of hand movements to compensate for the visuomotor rotation (Fig. 3C). The observed after-effect also suggests that the participants acquired implicit knowledge of the visuomotor rotation (Fig. 4B). These findings are in agreement with previous studies using a 30° rotation [60]–[61]. However, the participants did not acquire explicit knowledge of the rotation (Fig. 4C). Taken together, the participants adjusted the hand movements through both the visual feedback control and a use of implicit knowledge of the visuomotor rotation. As a result, movement time and trajectory length at the end of practice became similar to the baseline performance. These behavioral patterns reflect the relative ease of dealing with a small visuomotor rotation compared with greater ones [18], [27]–[29].

Regarding the 75° group, the magnitude of after-effect (−16°, Fig. 4E) was similar to that of the previous study [31], indicating that this group acquired much smaller amounts of implicit knowledge of the visuomotor rotation relative to the applied rotation. However, the initial direction error was unchanged with practice (Fig. 3C), which was different from other studies using 60°–90° rotations [21], [31], [42], [59]. Our further analysis revealed that the unchanged initial direction error was caused by a subgroup of the participants increasing the direction error during practice, while the other subgroup decreased it (see Appendix 1 in Text S1 and Fig. S1A). Furthermore, the curvature of hand paths (Appendix 1 – Fig. S1B) was much greater for the former subgroup than for the latter subgroup. Even the latter subgroup still had greater curvature than the 30° and 150° groups. Taken together, these results indicate that implicit knowledge of the curved hand paths acquired during the rotated environment resulted in a small after-effect without a substantial reduction of the initial direction error. A formation of smoothly curved hand paths through practice as well as its effectiveness under a 90° rotated environment were indeed reported previously [62], although another study did not report such curved hand paths [28]. Exact reasons for the discrepancies for the occurrence of curved hand paths are unknown. Large visuomotor rotations are more difficult than the small ones, thereby likely leaving room for applying diverse adaptive strategies among participants. Such diversity in the application of explicit adjustments was found previously [31].

For the 75° group, the value of adaptive shift (−27°, Fig. 4D) was far below the value that would fully compensate for the rotation (−75°). This indicates that the on-line feedback control was used to a great extent. Such extensive use of the feedback control was also observed previously for complex visuomotor transformations under on-line visual feedback, which resulted in limited acquisition of implicit knowledge of the transformations [43], [60]. Probably due to that and the curved hand paths, the movement time and the trajectory length at the end of practice never reached the same level as the baseline performance. Poor and inconsistent acquisition of explicit knowledge of the visuomotor rotation (Fig. 4F) and smaller amount of adaptive shifts, which differed from a previous study [31], may have been caused by the same factors.

Adaptation to the 150° rotation is thought to involve a two-step rotational transformation: a reversal shift, followed by a backward (30°) rotation (see Introduction). The 150° group had explicit knowledge of the visuomotor rotation at about −180° (Fig. 4I). As adaptive shift was about −163° (Fig. 4G), the rotational compensation excluding that from the on-line feedback control was not entirely attributed to a reversal shift based on the explicit knowledge. At the same time, this group had no implicit knowledge of the visuomotor rotation (Fig. 4H). These results suggest that an initial reversal shift was carried out as an explicit adjustment of hand directions, whereas the second step of a backward rotational transformation was accomplished mainly through the on-line feedback control instead of implicit learning. Interestingly, the participants produced a substantial variability in the initial direction error across trial blocks (Fig. 3C). A further analysis revealed that this group had greater inter-trial variability of this parameter within each participant than the other groups (Appendix 2 in Text S1 and Fig. S1D), suggesting the difficulty of precisely preplanning the aiming direction. This likely reflects the complexity of visuomotor processes involving the two-step transformation. Possibly for the same reason, movement time and trajectory length at the end of practice never reached the same level as that of the baseline.

In summary, the present results indicate that by and large, each of the three groups used a different control strategy for adapting their hand movements to the rotated environment. The 30° group used proportionately more implicit adjustments of movement directions. The 75° group used more of an on-line feedback control, while the 150° group used explicit strategic adjustments of movement directions.

### Adaptive changes of eye movements

A gaze anchoring behavior, i.e., gazing at the target during the hand movement, is commonly observed for goal-directed manual actions in a normal environment. Processing of retinal and extraretinal information due to gazing at the target not only improves the registration of target locations, but also planning and control of aiming movements (see Introduction). Accompanying adaptive changes of hand movements, the onset of gaze anchoring to the target vicinity was gradually shifted to an earlier phase of hand movements in all groups (Fig. 5). The gradual emergence of this gaze pattern is consistent with the study by Sailer et al. [17] where participants had to discover a complex, novel sensorimotor mapping rule between manual movements and cursor motions. Compared to that study, the current study required less exploration to discover a basic visuomotor mapping rule and mainly required adaptation of an existing visuomotor map to a rotated environment. Nevertheless, the gaze anchoring to the target was diminished at the initial encounter with the rotated environment, and it was gradually restored after repeated practice. This finding has two implications. First, the oculomotor system easily abandons the gaze anchoring pattern during hand movements when the cursor goes to unexpected directions compared to the existing mapping rule. Second, the oculomotor system establishes the gaze anchoring as the relation between the cursor and hand movements becomes more familiar, and thus more predictable. With that predictability in place, direction and amplitude of hand movements capable of bringing the cursor near the target can be planned and executed; and importantly, such an outcome can be predicted. Based on this prediction, the oculomotor system can anchor the gaze to the target in the early phase of hand movement and prepare for the next control event (i.e., homing in the cursor to the target). Therefore, the establishment of gaze anchoring reflects a functional change of gaze control, namely from a reactive control for assessing an unexpected environment to a predictive control for guiding the hand to an action goal [17], [63].

The results of gaze locations prior to the gaze anchoring revealed that all groups mostly looked at the cursor area compared to other areas in the work space at the early practice phase (Fig. 6). The gaze fixation to the cursor area may be useful to explore the relation between the cursor and hand movements because a foveal vision on the cursor can provide more precise perception of cursor locations and the directions of cursor movements [17], [64], [65]. This, in turn, provides more precise estimates of error signals between the cursor and hand movements, thereby helping to plan and adjust the directions of hand movements that would bring the cursor to the direction of the target.

The duration of gazing at the cursor area was gradually decreased with practice (Fig. 6E), whereas the gaze duration at the mid area between the starting position and the target was increased from the early practice phase (Fig. 6C). A similar pattern of the mid-area gazing was also observed by Sailer et al. [17] during an intermediate learning phase following a long initial exploratory phase. The authors interpreted the mid-area gaze pattern as reflecting a compromise between having parafoveal/foveal vision near the cursor and fixating on a useful goal to guide the cursor in the direction of the target, or setting a more manageable preliminary goal for the cursor. Supporting this interpretation, other studies that examined effects of various prescribed gaze locations on learning of a visuomotor transformation revealed the importance of processing both the target and cursor information [66], [67]. Taking these aspects together, the increased duration of the mid-area gazing is likely associated with the sensorimotor processing of both the target and cursor information to guide the cursor to the direction of the target, thereby serving as an intermediate step toward establishing a gaze anchoring behavior to the target.

The detection of errors between the cursor and the target as well as errors between the cursor and the planned hand movements are thought to be important for learning of visuomotor transformations [44], [68], [69]. Both implicit and explicit processes are thought to work in parallel to reduce these errors [44], [70]–[72], while affecting adaptive behavior differently. The present results of hand movements reveal that the involvement of these processes varies depending on the rotational magnitudes. Interestingly, previous studies showed that visuomotor adaptation based on the implicit process resulted in better adaptive behavior (e.g., faster and less variable movements [33]) with stronger or long-lasting after-effects [33], [69], [73]–[75] than the adaptation that involved the explicit process as well. The present results of gaze patterns further extend our knowledge by revealing that the above errors are detected by placing the cursor in the foveal/parafoveal vision at the early practice phase but in the peripheral vision at the later phase. The gaze anchoring pattern in the later practice phase is considered useful because it places the visual target in fovea as the cursor approaches the target, while temporally removing the added burden of spatial updating for gaze shift [65], [76]. Thus, by having a spatial coordinate of the eyes aligned on the task goal, the sensorimotor system may be able to better estimate these errors relative to the rotated sensorimotor map, thereby contributing to learning of visuomotor transformations.

It is important to note that the present results suggest a greater generality of the adaptive pattern (i.e., establishing gaze anchoring to the target) implemented by the oculomotor system compared to more diverse control strategies implemented by the limbmotor system for manual actions during a visuomotor adaptation (such as the use of a feedback control, implicit adjustments of movement directions, and explicit strategic adjustments). As discussed above, the key factor for the oculomotor system to trigger gaze anchoring seems to be a high predictability that the upcoming ballistic hand movement will bring the cursor near the target under the rotated environment. This triggering rule is likely applicable regardless of which control strategies are used to produce such hand movement. This generality of adaptive gaze patterns may reflect the role of gaze control as an integral but general component of the planning and control mechanisms that implement various control strategies for goal-directed manual actions.

Based on the current study, however, it is difficult to determine whether establishing gaze anchoring to the target plays a direct functional role for implementing each of those adaptive strategies employed by the limbmotor system. Our most recent study showed that the utilization of explicit strategic adjustments under a visuomotor rotation was altered by whether gaze was fixed to the target or the hand target (where the hand had to reach for counteracting the rotated visual feedback) [77]. Other studies also reported that fixating gazes on the target alone throughout practice was detrimental to a visuomotor adaptation [66]–[67]. These studies suggest that adaptive changes of gaze behavior play functional roles for visuomotor adaptation. Further exploration of this issue is an exciting direction of future studies.

### Rotation-specific gaze patterns

Aside from the above general changes of gaze patterns, we observed some specific changes depending on the magnitude of the visuomotor rotation. Compared to other groups, the 30° group made an earlier onset of gaze anchoring to the target vicinity during hand movements at the beginning of practice (Fig. 5). Thus, the function of eye movements seems to be more focused on guiding the hand toward the target rather than exploring the relation between the cursor and hand movements. This gaze pattern likely reflects a relative ease of adapting to a small visuomotor rotation as discussed above. In contrast, the 75° group spent a longer time to gaze at the cursor area (Fig. 6E, F) and delayed the establishment of gaze anchoring during practice (Fig. 5). Hence, this group clearly needed a longer exploration period to find the cursor-hand relation than the 30° group.

Only the 150° group failed to restore an onset timing of gaze anchoring in the late practice phase similar to that of the baseline performance (Fig. 5). This group persisted in gazing at the cursor and the starting position areas prior to the gaze anchoring (Fig. 6E, G, Fig. 7). These gaze patterns suggest that the participants needed to carefully plan the strategic adjustments of hand movements for a reversal shift and verify the progress of cursor movements from the movement onset. Furthermore, to accomplish a subsequent backward rotational adjustment, on-line visual assessments of cursor locations were needed for further planning and adjustments of the hand movements to redirect the cursor toward the target. These two processes must have delayed the establishment of gaze anchoring to the target. Moreover, the on-line visual assessment of cursor locations was likely enhanced in this group because of the observed difficulty in precisely planning the initial direction of hand movements (Fig. 3C, Appendix 2 – Fig. S1D). Interestingly, previous studies implied that applying such two-step transformation for 150° is easier than a single-step transformation with a large rotation, such as 90° [18], [27], [28]. Our results of overall hand performance (i.e., movement time and trajectory length) support this postulate. However, gaze patterns assisting such a two-step process for manual actions turned out to be different compared to those of a single-step process.

The current study tested only three rotation angles (30°, 75°, and 150°). However, further examinations of other rotation angles will provide better understanding of the proportional effect of rotation size on implicit/explicit processes and gaze anchoring behavior. For example, testing the 180° rotation will clarify the formation of gaze anchoring for the visuomotor transformation where an explicit process (a reversal shift) is mainly utilized. A recent study showed that saccadic patterns to the target during the 180° rotation were more stable than that during the 90° rotation [45]. Testing other rotation angles, such as ones between 30° and 75° and between 75° and 150°, will further clarify a proportional shift between implicit and explicit processes and variations of the two-step transformation.

In summary, the present results support two conclusions: (1) that gaze patterns during aiming action change from the early to late practice phase of a visuomotor transformation, namely from gazing at the feedback cursor to gazing at the target, and (2) that the speed of this adaptive change and the proportion of using these two gaze patterns are altered depending on the difficulty of visuomotor transformations. The adaptive changes of the gaze patterns reflect a functional change of gaze control from a reactive control for exploring the relation between the cursor and hand movements to a predictive control for guiding the hand to the task goal. This functional change contributes to planning and control mechanisms that implement various control strategies for goal-directed manual actions.

## Supporting Information

Figure S1 **Adaptive changes of initial direction error (A), curvature of hand trajectory (B), hand trajectory length in the pre-gaze anchoring (Pre-GA) period (C), and SD of initial direction error during practice of a visuomotor rotation.** Mean values of all participants are plotted against 40 trial blocks with 4 trials for the 30° (white diamonds in B and D), 75° (black circles in D), and 150° (grey squares in B and D) groups as well as subgroups of the 75° group (IDE-Small: black circles; IDE-Large: white circles in A, B, and C). Mean values from the baseline condition (BL) are also plotted. In C, the values are expressed as a percentage of the total trajectory length. The error bars represent standard errors.
(PDF)

Text S1 **Appendix 1 and Appendix 2.**
(PDF)
