# Supplementary material for: Eye-Hand Coordination during Visuomotor Adaptation with Different Rotation Angles
Source: PLoS One. 2014 Oct 15;9(10):e109819. doi: 10.1371/journal.pone.0109819 (PMC4198129; doi:10.1371/journal.pone.0109819)
Supplement: Figure S1 — Adaptive changes of initial direction error (A), curvature of hand trajectory (B), hand trajectory length in the pre-gaze anchoring (Pre-GA) period (C), and SD of initial direction error during practice of a visuomotor rotation. Mean values of all participants are plotted against 40 trial blocks with 4 trials for the 30° (white diamonds in B and D), 75° (black circles in D), and 150° (grey squares in B and D) groups as well as subgroups of the 75° group (IDE-Small: black circles; IDE-Large: white circles in A, B, and C). Mean values from the baseline condition (BL) are also plotted. In C, the values are expressed as a percentage of the total trajectory length. The error bars represent standard errors. (PDF) [file pone.0109819.s001.pdf]

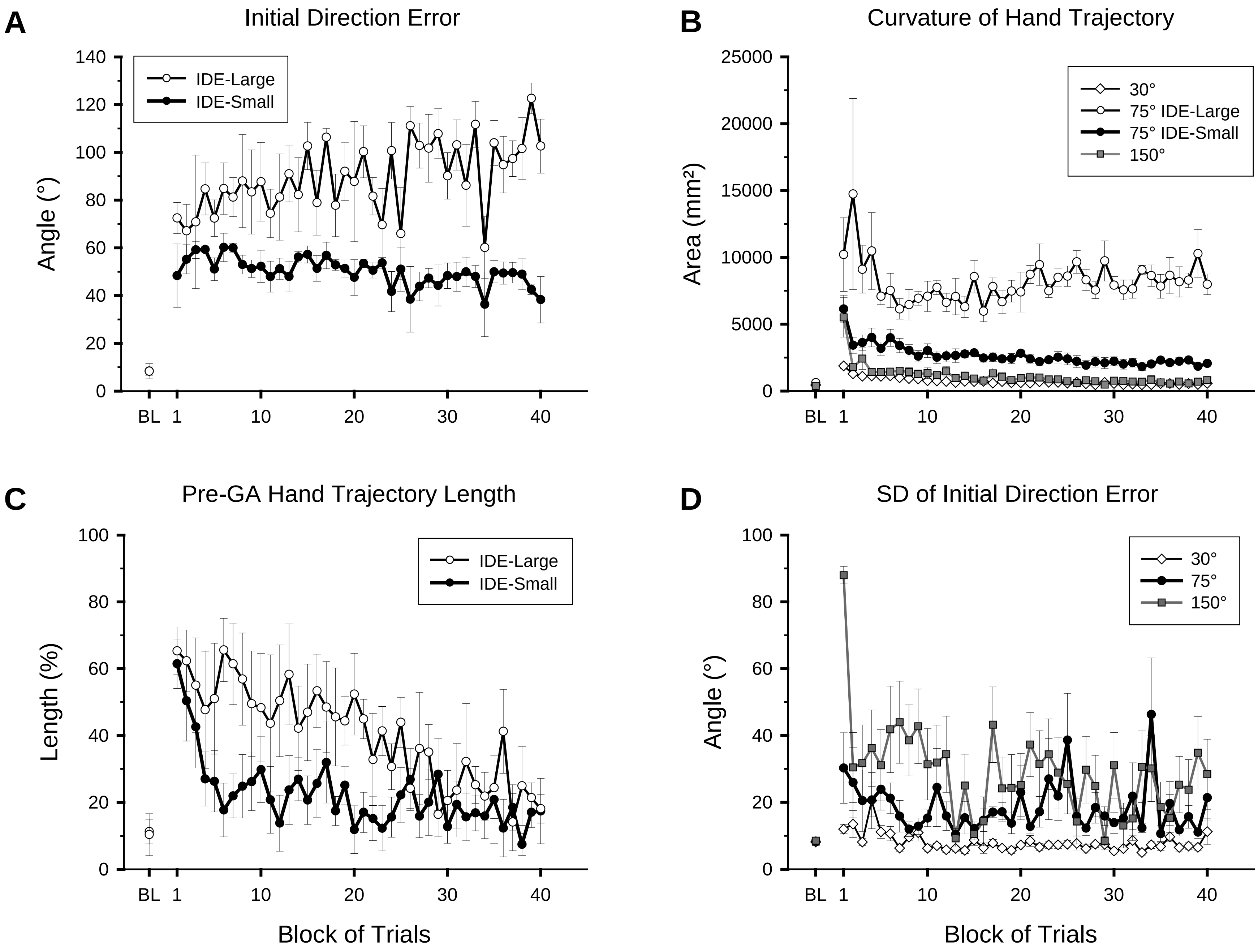

A
Initial Direction Error
IDE-Large
IDE-Small
Angle (°)
0
20
40
60
80
100
120
140
BL
1
10
20
30
40
B
Curvature of Hand Trajectory
30°
75° IDE-Large
75° IDE-Small
150°
Area (mm²)
0
5000
10000
15000
20000
25000
C
Pre-GA Hand Trajectory Length
Length (%)
D
SD of Initial Direction Error
30°
75°
150°
Angle (°)
Block of Trials
Block of Trials

Figure S1. Adaptive changes of initial direction error (A), curvature of hand trajectory (B), hand trajectory length in the pre-gaze anchoring (Pre-GA) period (C), and SD of initial direction error during practice of a visuomotor rotation. Mean values of all participants are plotted against 40 trial blocks with 4 trials for the 30° (white diamonds in B and D), 75° (black circles in D), and 150° (grey squares in B and D) groups as well as subgroups of the 75° group (IDE-Small: black circles; IDE-Large: white circles in A, B, and C). Mean values from the baseline condition (BL) are also plotted. In C, the values are expressed as a percentage of the total trajectory length. The error bars represent standard errors.
