# Supplementary material for: Eye-Hand Coordination during Visuomotor Adaptation with Different Rotation Angles
Source: PLoS One. 2014 Oct 15;9(10):e109819. doi: 10.1371/journal.pone.0109819 (PMC4198129; doi:10.1371/journal.pone.0109819)
Supplement: Text S1 — Appendix 1 and Appendix 2. (PDF) [file pone.0109819.s002.pdf]

APPENDIX 1

Initial direction error in the 75° group

Mean initial direction error of the 75° group was not reduced during the practice condition (Fig. 3C, black circles), which was inconsistent with previous studies using 60°-90° rotations [21,31,42,59]. To explore the reasons for this discrepancy, we classified the participants based on the size of their initial direction error in the late practice phase into two subgroups: one with small initial direction errors < 60° (IDE-Small, n = 6); and the other with large errors > 90° (IDE-Large, n = 4). Mean initial direction error for each subgroup is shown in Figure S1A. IDE-Small participants significantly reduced the error from the early (54.2°) to the late practice phase (43.2°, paired t-test, $t(5) = 2.66$, $p < 0.05$), even though the latter value was still far greater than the baseline value (8.4°). In contrast, IDE-Large participants significantly increased the error from the early (70.2°) to the late practice phase (108.9°, $t(3) = 7.81$, $p < 0.01$). Thus, a non-reduction of the initial direction error found in the 75° group was caused by inter-individual variability that resulted in the two opposite adaptive patterns during the course of practice.

There was a possibility that the 75° group adapted to use curved hand paths [62], thereby contributing the above results of initial direction error. Thus, we have examined the curvature of hand path by measuring the absolute value of integrated area between hand trajectory and the straight line connecting the start position and the target. Mean curvature across participants is shown in Figure S1B. IDE-Large participants increased the curvature at the beginning of practice compared to the baseline condition, but decreased it until the 7th trial block. Thereafter, the curvature was gradually increased. The difference between the early and late phases was not significant (paired t-test, $p > 0.05$). In contrast, IDE-Small participants significantly decreased the curvature from the early to the late practice phase (paired t-test,

$t(5) = 5.30$, $p < 0.01$). In the late practice phase, the mean value across IDE-Large participants was significantly greater than that across IDE-Small participants (independent t-test, $t(8) = 9.39$, $p < 0.001$). Thus, IDE-Large participants used more curved hand paths than the IDE-Small participants. Note that even for the IDE-Small participants, mean curvature in the late phase was significantly greater than those of the 30° (independent t-test, $t(12) = 11.54$, $p < 0.001$) and the 150° groups ($t(14) = 8.15$, $p < 0.001$). Hence, hand paths of IDE-Small participants were more curved than those of the 30° and 150° groups.

Finally, we examined if adaptive changes of eye movements differed between IDE-Small and IDE-Large participants. Regarding the establishment of gaze anchoring to the target, mean pre-gaze anchoring trajectory lengths were increased from the baseline condition to the early practice phase in both subgroups (Fig. S1C). There was no subgroup difference in this phase (independent t-test, $p > 0.05$). Both subgroups reduced the trajectory lengths throughout practice. However, IDE-Small participants did so more rapidly than IDE-Large participants. At the end of practice, the trajectory lengths did not differ between the two groups (independent t-test, $p > 0.05$). These results indicate that both subgroups established gaze anchoring by the end of practice, but IDE-Small participants established it much faster than the IDE-Large participants. Additionally, in terms of gaze patterns during the pre-gaze anchoring period, the average distances between the cursor and gaze location (the parameter shown in Fig. 7) were similar between these subgroups.

APPENDIX 2

Inter-trial variability of initial direction error

Mean initial direction error of the 150° group substantially fluctuated around 0º during practice (Fig. 3C, grey squares). This phenomenon was possibly caused by large inter-individual variability and intra-individual variability. To examine whether the intra-individual

variability differed among the three rotation groups, we measured SD of initial direction error across trials in each practice trial-block for each participant. Mean values of the individual SDs are plotted in Figure S1D. A 3 (group: 30°, 75°, and 150°) x 2 (phase: early and late practice) ANOVA revealed that the 150° group had significantly greater intra-individual variability than the other groups ($F(2,25) = 13.63$, $p < 0.001$, post hoc: $p < 0.01$ for both comparisons), which did not differ from each other ($p > 0.05$). The intra-individual variability was significantly reduced from the early to late practice phase ($F(1,25) = 12.19$, $p < 0.01$). There was no group-by-phase interaction. The observed large intra-individual variability of initial direction error in the 150° group suggests a difficulty to preplan hand directions under the 150° rotation. This partially explains the large variability of the initial direction error across trial blocks found in this group (Fig. 3C).
